# COVID-19: Famotidine, Histamine, Mast Cells, and Mechanisms

**DOI:** 10.3389/fphar.2021.633680

**Published:** 2021-03-23

**Authors:** Robert W. Malone, Philip Tisdall, Philip Fremont-Smith, Yongfeng Liu, Xi-Ping Huang, Kris M. White, Lisa Miorin, Elena Moreno, Assaf Alon, Elise Delaforge, Christopher D. Hennecker, Guanyu Wang, Joshua Pottel, Robert V. Blair, Chad J. Roy, Nora Smith, Julie M. Hall, Kevin M Tomera, Gideon Shapiro, Anthony Mittermaier, Andrew C. Kruse, Adolfo García-Sastre, Bryan L. Roth, Jill Glasspool-Malone, Darrell O. Ricke

**Affiliations:** ^1^ RW Malone MD LLC, Madison, VA, United States; ^2^ Medical School Companion LLC, Marco Island, FL, United States; ^3^ MIT Lincoln Laboratory, Lexington, MA, United States; ^4^ Department of Pharmacology, University of North Carolina, Chapel Hill, Chapel Hill, NC, United States; ^5^ Department of Microbiology, Icahn School of Medicine at Mount Sinai, New York, NY, United States; ^6^ Global Health and Emerging Pathogens Institute, Icahn School of Medicine at Mount Sinai, New York, NY, United States; ^7^ Department of Biological Chemistry and Molecular Pharmacology, Blavatnik Institute, Harvard Medical School, Boston, MA, United States; ^8^ Department of Chemistry, McGill University, Montreal, QC, Canada; ^9^ Molecular Forecaster Inc, Montreal, QC, Canada; ^10^ Tulane National Primate Research Center, Covington, LA, United Sates; ^11^ Department of Pathology and Laboratory Animal Medicine, Tulane University School of Medicine, New Orleans, LA, United States; ^12^ Department of Microbiology and Immunology, Tulane University School of Medicine, New Orleans, LA, United States; ^13^ Frank H. Netter MD School of Medicine – Quinnipiac University, Hamden, CT, United States; ^14^ Department of Urology, Beloit Memorial Hospital, Beloit, WI, United States; ^15^ Pharmorx LLC, Gainesville, FL, United States; ^16^ Division of Infectious Diseases, Department of Medicine, Icahn School of Medicine at Mount Sinai, New York, NY, United States; ^17^ Icahn School of Medicine at Mount Sinai, The Tisch Cancer Institute, New York, NY, United States

**Keywords:** famotidine (PubChem CID: 3325), mast cell activating disorder, COVID-19, hyperinflammation state, GPCR (G Protein Coupled Receptors), histamine (H2) receptor

## Abstract

SARS-CoV-2 infection is required for COVID-19, but many signs and symptoms of COVID-19 differ from common acute viral diseases. SARS-CoV-2 infection is necessary but not sufficient for development of clinical COVID-19 disease. Currently, there are no approved pre- or post-exposure prophylactic COVID-19 medical countermeasures. Clinical data suggest that famotidine may mitigate COVID-19 disease, but both mechanism of action and rationale for dose selection remain obscure. We have investigated several plausible hypotheses for famotidine activity including antiviral and host-mediated mechanisms of action. We propose that the principal mechanism of action of famotidine for relieving COVID-19 symptoms involves on-target histamine receptor H_2_ activity, and that development of clinical COVID-19 involves dysfunctional mast cell activation and histamine release. Based on these findings and associated hypothesis, new COVID-19 multi-drug treatment strategies based on repurposing well-characterized drugs are being developed and clinically tested, and many of these drugs are available worldwide in inexpensive generic oral forms suitable for both outpatient and inpatient treatment of COVID-19 disease.

## Introduction

Severe Acute Respiratory Syndrome Coronavirus 2 (SARS-CoV-2) is a highly infectious and pathogenic betacoronavirus first detected in human infections during December 2019 (Wu D. et al., 2020; Wu and McGoogan, 2020; Zhu et al., 2020). Coronavirus infectious disease—2019 (COVID-19) is a disease spectrum causally associated with infection by SARS-CoV-2. Definitive COVID-19 diagnosis requires the presence of the virus, which can be isolated, grown, or otherwise detected as unique SARS-CoV-2 viral nucleic acid sequences. There are SARS-CoV-2 virus shedding or nucleic acid positive patients that do not manifest clinical COVID-19 (Danis et al., 2020; Furukawa et al., 2020; Ki and Task Force for -nCo, 2020; Lai et al., 2020; Pan et al., 2020; Zou et al., 2020). 13–20% of patients with symptoms develop severe respiratory compromise requiring oxygenation, with radiological findings of ground glass opacities and consolidation (Huang et al., 2020; Shi et al., 2020; Wang et al., 2020). Up to 80% of SARS-CoV-2 infected patients are asymptomatic and do not appear to progress to COVID-19 (Tian et al., 2020a; BMJ, 2020; Day, 2020; He et al., 2020; Hu et al., 2020; Mizumoto et al., 2020; WHO, 2020). Therefore, SARS-CoV-2 infection is necessary but not sufficient for development of clinical COVID-19 disease.

Patients with COVID-19 disease can present with a range of mild to severe non-specific clinical signs and symptoms which develop 2–14 days after exposure to SARS-CoV-2. These symptoms include cough or shortness of breath, and at least two of the following; fever, chills, repeated rigor, myalgia, headache, oropharyngitis, anosmia, and ageusia (Giacomelli et al., 2020; Lechien et al., 2020). More severe symptoms warranting hospital admission include difficulty breathing, a persistent sense of chest pain or pressure, confusion or difficulty to arouse, and central cyanosis. Of hospitalized patients, 20–42% develop Acute Respiratory Disease Syndrome (ARDS), the most common cause for admission to hospital intensive care units (ICU). 39–72% of patients admitted to the ICU will die (CDC, 2020).

Clinical data from a variety of sources indicate that famotidine treatment may reduce morbidity and mortality associated with COVID-19, but other studies suggest no clinical benefits from famotidine treatment. An early retrospective cohort study of 1,620 hospitalized COVID-19 patients indicates that 84 propensity score matched patients receiving famotidine during hospitalization (oral or IV, 20 mg or 40 mg daily) had a statistically significant reduced risk for death or intubation (adjusted hazard ratio (aHR) 0.42, 95% CI 0.21–0.85) and also a reduced risk for death alone (aHR 0.30, 95% CI 0.11–0.80) (Freedberg et al., 2020). More recent retrospective studies concerning famotidine and COVID-19 have been more variable and generally less sanguine, ranging from no effect (Cheung et al., 2020; Shoaibi et al., 2020; Yeramaneni et al., 2020) to effects similar to those originally reported (Odds Ratio—OR-death 0.37, OR-death or intubation 0.47) (Mather et al., 2020). The difference in measured outcomes between the two most recent studies reporting no effect (Shoaibi et al., 2020; Yeramaneni et al., 2020) and the study from Hartford hospital (Mather et al., 2020) may have multiple explanations, including famotidine dosage. An early limited case series suggested that it is necessary to administer famotidine at higher than standard over-the-counter doses to relieve COVID-19 symptoms (ergo, high dose [HD] famotidine treatment) (Janowitz et al., 2020). Together, these data have been interpreted as indicating that the increased survival pattern initially reported (Freedberg et al., 2020) is due to an off-target, non-histamine receptor-mediated property of famotidine not shared with cimetidine (Borrell, 2020; Freedberg et al., 2020; Janowitz et al., 2020). HD famotidine was initially being tested in the United States (US) under an Investigational New Drug (IND) waiver for treating COVID-19. This early double blind randomized clinical trial involved high intravenous famotidine doses in combination with either hydroxychloroquine or remdesivir (ClinicalTrials.gov Identifier: NCT04370262), but that trial has been delayed and is yet to complete enrollment or report results. HD famotidine for treatment of COVID-19 is now also being clinically tested in Bangladesh (ClinicalTrials.gov Identifier: NCT04504240), and Iran (Samimagham et al., 2020). Recent encouraging clinical reports include simultaneous administration of famotidine and cetirizine at standard OTC doses (Hogan et al., 2020) and HD famotidine with celecoxib (Tomera and Kittah, 2020a; Tomera and Kittah, 2020b).

Herein we aim to investigate how famotidine may act to relieve early phase COVID-19 clinical symptoms. The most likely mechanisms of actions include: *via* antiviral activity, via novel human targets, or via the on-target mechanism described in the current FDA market authorization—famotidine is a histamine receptor H_2_ antagonist (and inverse agonist).

## Methods

### Experimental Analysis of the Mechanism of Action of Famotidine

Famotidine was originally selected by the authors for advancement as a potential repurposed drug candidate therapeutic for COVID-19 based on molecular docking data to the SARS-CoV-2 papain-like protease (PLpro). Based on this analysis the US Food and Drug Administration (FDA) granted an IND waiver for the subsequent double blinded randomized clinical trial currently in progress (ClinicalTrials.gov Identifier: NCT04370262). Briefly, a ranked list of licensed compounds with predicted binding activity in the PLpro catalytic site was computationally generated, and the PLpro catalytic site binding pose of each of the top compounds was examined and ranked by a team of pharmaceutical chemists. Package inserts or product monographs for the licensed compounds which generated high computational binding scores and passed inspection were then reviewed and used to rank compounds based on adverse events, FDA warnings, drug interactions on-target mechanisms, pharmacokinetic and absorption, metabolism, excretion and toxicity (ADMET), protein binding and available therapeutic window considerations. Famotidine (“PEPCID®”), a histamine H_2_ antagonist (inverse agonist) widely available in tablet form over-the-counter, as well as in solution form for intravenous administration, was repeatedly computationally ranked as among the most promising of the compounds tested and was associated with the most favorable pharmacokinetic and safety profile.

Recognizing that computational docking predictions are typically associated with about a 20% success rate, we applied the method of multiple working hypotheses (1890) to assess the mechanism of action of famotidine as a potential treatment for COVID-19. Relevant hypotheses tested included;

• Direct binding and action as an inhibitor of SARS-CoV-2 PLpro.
• Action as a direct acting inhibitor of SARS-CoV-2 infection or replication.
• Off-target binding and inhibition of either sigma receptors or a non-histamine H_2_ G-coupled protein receptor.
• Histamine H_2_ receptor inhibition.

Initial lay press journalistic reports based on unverified hearsay from the People’s Republic of China (PRC) (Borrell, 2020) suggested that famotidine provided clinical benefit for treatment of COVID-19, but other H_2_ receptor antagonists (cimetidine) did not. This resulted in an inference that famotidine did not act *via* its known mechanism of action as an H_2_ receptor inhibitor. Pharmacokinetic analyses were performed to model systemic circulating levels of famotidine and cimetidine at various doses.

If famotidine relieves clinical symptoms of COVID-19, and acts via known inhibitory and inverse agonist interactions with H_2_ receptors, then there must be a histopathological source of histamine release in damaged tissues including peripheral lung. One of the most common cellular sources of histamine are mast cells, so SARS-CoV-2 was used to experimentally infect African green monkeys (AGM). At necropsy, AGM lung sections from diseased and control lung parenchyma were sampled and stained for presence and density of mast cells.

### Does Famotidine Directly Bind and Act as an Inhibitor of SARS-CoV-2 PLpro?

#### Production of Recombinant SARS-CoV-2 Plpro

An expression plasmid containing the sequence for (His)6-TEVsite-SARS-CoV-2 PLpro (nsp3 from Wuhan-Hu-1 isolate, polyprotein 1ab 1564-1878) was obtained commercially from ATUM. The plasmid was transformed into *E. coli* BL21 (DE3) pLysS. The expression and purification protocols were adapted from prior work (Lindner et al., 2005).

#### Production of Recombinant ISG15

The expression plasmid for proISG15 (2-165) was a gift from David Komander (Addgene plasmid #110762; http://n2t.net/addgene:110762 ; RRID:Addgene_110762) (Swatek et al., 2018). Expression and purification protocols were adapted from (Swatek et al., 2018). A size exclusion chromatography step on a Superdex 75 column (GE Healthcare) was added as a final step.

#### PLpro Activity Assays

Cleavage of ISG15 by SARS-CoV-2 PLpro was tested by incubating 4 nM of PLpro in 50 mM Tris-HCl (pH 7.3), 150 mM NaCl, 2 mM DTT, 0.1 mg ml^−1^ BSA, with 10 µM of ISG15 in a final volume of 20 µL for 1 h at room temperature. Control was incubated without enzyme. Samples were subjected to SDS-PAGE.

### Does Famotidine Directly Inhibit SARS-CoV-2 Infection or Replication in Vero Cells?

#### Viral Growth and Cytotoxicity Assays in the Presence of Inhibitors

2,000 Vero E6 cells were seeded into 96-well plates in Dulbecco’s Modified Eagles Medium (DMEM, 10% FBS) and incubated for 24 h at 37°C, 5% CO_2_. Two hours before infection, the medium was replaced with 100°ul of DMEM (2% FBS) containing the compound of interest at concentrations 50% greater than those indicated, including a DMSO control. Plates were then transferred into the Biosafety Level 3 (BSL3) facility and 100 PFU (MOI 0.025) was added in 50°ul of DMEM (2% FBS), bringing the final compound concentration to those indicated. Plates were then incubated for 48 h at 37°C. After infection, supernatants were removed and cells were fixed with 4% formaldehyde for 24 h prior to being removed from the BSL3 facility. The cells were then immunostained for the viral NP protein with a DAPI counterstain. Infected cells (488 nM) and total cells (DAPI) were quantified using the Celigo (Nexcelcom) imaging cytometer. Percent infection was quantified as ((Infected cells/Total cells) − Background) *100 and the DMSO control was then set to 100% infection for analysis. The IC_50_ and IC_90_ for each experiment were determined using the Prism (GraphPad Software) software. For select inhibitors, infected supernatants were assayed for infectious viral titer using the TCID_50_ method. Cytotoxicity was also performed using the MTT assay (Roche), according to the manufacturer’s instructions. Cytotoxicity was performed in uninfected Vero E6 cells with same compound dilutions and concurrent with viral replication assay.

#### TCID50 Assay

Infectious supernatants were collected at 48 h post infection and frozen at −80°C until later use. Infectious titers were quantified by limiting dilution titration on Vero E6 cells. Briefly, Vero E6 cells were seeded in 96-well plates at 20,000 cells/well. The next day, SARS-CoV2-containing supernatant was applied at serial 10-fold dilutions ranging from 10^−1^ to 10^−6^ and, after 5 days, viral CPE was detected by staining cell monolayers with crystal violet. Median tissue culture infectious doses (TCID50)/ml were calculated using the method of Reed and Muench.

### Does Famotidine Bind and Interact with the Sigma 1 or 2 Receptors?

#### Sigma-1 and Sigma-2 Competition Binding Assays

Sigma-1 receptor [^3^H](+)-pentazocine competition curves testing the binding of Famotidine, Cimetidine, and PB-28 (as positive control), were performed with Expi293 cells (Thermo Fisher) overexpressing the human sigma-1 receptor. Membranes were incubated in a 100 μL reaction buffered with 50 mM Tris (pH 8.0), with 10 nM [3H](+)-pentazocine, 0.1% BSA, and seven concentrations (ranging from 10 μM to 0.1 nM) of the competing cold ligand. Reactions were incubated for 2 h at 37°C and then were terminated by filtration through a glass fiber filter using a Brandel cell harvester. Glass fiber filters were soaked in 0.3% polyethylenimine for at least 30 min at room temperature before harvesting. All reactions were performed in triplicate using a 96-well block. After the membranes were transferred to the filters and washed, the filters were soaked in 5 ml Cytoscint scintillation fluid overnight, and radioactivity was measured using a Beckman Coulter LS 6500 scintillation counter. Data were analyzed using GraphPad Prism software. Ki values were computed by directly fitting the data and using the experimentally determined probe Kd to calculate a Ki value, using the GraphPad Prism software. This process implicitly uses a Cheng–Prusoff correction, so no secondary correction was applied.

Sigma-2 competition curves were performed in a similar manner, using Expi293 cells overexpressing the human sigma-2 (TMEM97) and using [^3^H] DTG as the radioactive probe.

### Does Famotidine Act via an Off-Target Activity Involving a G-Coupled Protein Receptor (GPCR) Other than the Histamine H_2_ GPCR?

Human G-coupled Protein Receptor genome (GPCRome) screening was carried out according to published procedure (Kroeze et al., 2015) with minor modifications. In brief, HTLA cells were subcultured into poly-L-lysine coated clear bottom 384-well white plates at a density of 6,000 cells/well in 40 ul of DMEM supplemented with 1% dialyzed FBS for overnight, transfected with 20 ng/well Tango constructs for 24 h, received drug stimulation (10 uM final) for another 24 h. Medium and drug solutions were removed and Bright-Glo Reagents (Promega) were added for luminescence counting. For concentration response assays, HTLA cells were transfected with Tango constructs for 24 h, plated in poly-L-Lysine coated clear bottom 384-well white plates at a density of 10,000 cells/well in 40 ul of DMEM supplemented with 1% dialyzed FBS for 6 h before receiving drugs for overnight stimulation. Plates were then counted as above.

### Does Famotidine Act by Blocking Histamine H_2_ receptor(s)?

The known on-target activity of famotidine considered the known primary mechanism of action is as an antagonist of the histamine H_2_ receptor. This hypothesis was originally rejected due to unverified reports that clinical researchers in PRC (Wuhan) had observed that famotidine use was associated with protection from COVID-19 mortality, while the histamine H_2_ receptor antagonist cimetidine was not. Positing that this difference in clinical effectiveness for the two different H_2_ receptor antagonists may reflect absorption, pharmacokinetic and pharmacodistribution differences between famotidine and cimetidine, steady state concentrations were calculated for both drugs when administered at standard oral doses as well as the elevated doses of famotidine which are being prescribed off-label for outpatient clinical use to treat COVID-19 or are being used in the ongoing inpatient clinical trial (NCT04370262), and these were compared to the published H_2_ receptor IC_50_ for each drug.

#### GloSensor cAMP Assays

cAMP production was determined in transiently transfected HEK293T cells (Stauch et al., 2019) with minor modifications. In brief, HEK293 T cells were co-transfected with H_2_ and GloSensor cAMP reporter DNA (Promega) overnight and plated in poly-l-lysine coated clear bottom 384-well white plate at a density of 15,000 cells/well in 40 ul of DMEM supplemented with 1% dialyzed FBS for 6 h. Medium was removed and cells were loaded with 3 mM luciferin in drug buffer (20 ul/well, 1x HBSS, 20 mM HEPES, pH 7.40) for 30 min. Test compounds were prepared in the same drug buffer supplemented with 1 mg/ml BSA and added (20 ul/well at 2x) to cells. Luminescence was counted after 20 min and results were analyzed in Prism 8.4.

Radioligand binding assays with human histamine receptors. Radioligand binding assays with human H_1_, H_2_, H_3_, and H_4_ receptors were conducted according to the NIMH PDSP assay protocol (https://pdsp.unc.edu/pdspweb/?site=assays) and published procedures (Besnard et al., 2012).

#### Famotidine and Cimetidine Pharmacokinetic Analyses

Steady-state values of famotidine and cimetidine at various doses were calculated as follows using standard pharmacokinetic calculations. Bioavailability, and clearance values were obtained from data reported by Lin (1991) for tablet and capsule dosing of famotidine and cimetidine; for 60 mg famotidine bid, kinetic data were obtained from a report by Yeh et al. (1987). For intravenous administration of famotidine (IV 120 mg q8 hours), kinetic values were obtained from the famotidine New Drug Applications (NDAs 19-510/S-029, 20-249/S-012).

Calculations: Area under the curve (AUC) determinations were made as follows: AUC (mg h/L) = (F x Dose)/Cl; where F = bioavailability, Cl = clearance. Steady (Css) state values were calculated as follows: Css (μg/L) = AUC/T; where T = dosing interval (h). Css levels were converted to μM using the molecular weights of famotidine (337.45) and cimetidine (252.34), respectively.

### Are Increased Numbers Mast Cells Present in Lung Parenchyma of SARS-CoV-2 Infected AGM?

African green monkeys were exposed by aerosol to SARS-CoV-2 and euthanized after progression of clinical signs or at the end of the study period (four weeks post-infection) (Blair et al., 2020). Sections of lung were stained with toluidine blue and digitally scanned with a Zeiss Axio Scan.Z1 slide scanner (Carl Zeiss Microscopy, White Plains, NY). Whole slide images were analyzed using the digital image analysis software, HALO Multiplex IHC v2.1.1 (Indica Labs, Albuquerque, NM). Mast cells were quantified by the detection of metachromasia within the cytoplasm. The positive threshold for the detection of metachromasia was set by a veterinary pathologist using a slide of a mast cell tumor as a positive control. Annotation regions were drawn around the entire lung section and the analysis was run on the annotation region. The proportion of mast cells over the total number of cells detected were reported.

## Results

### Analysis of the Mechanism of Action of Famotidine

#### Famotidine Does Not Bind to SARS-CoV-2 Proteases

**FIGURE 1:**
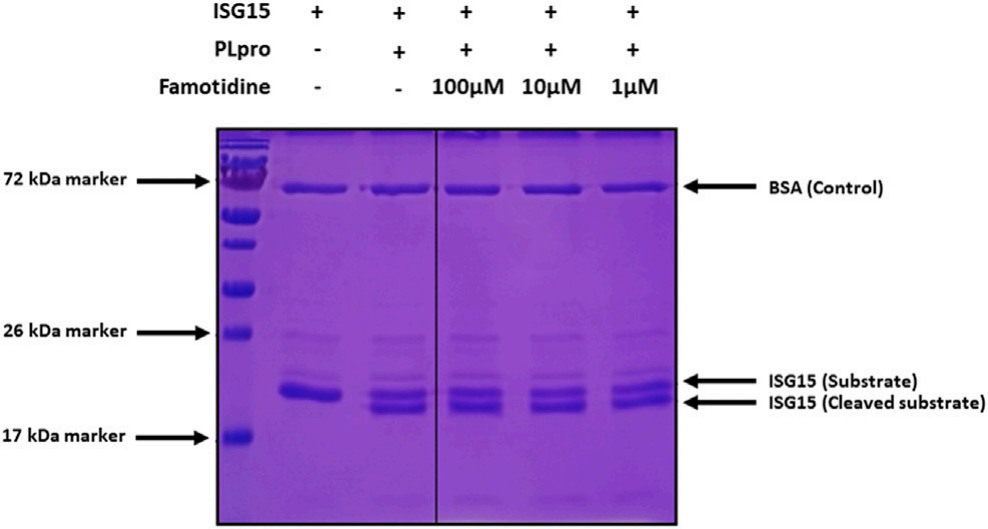
Cleavage of ISG15 C-terminal 8 amino acids by SARS-CoV-2 PLpro purified from *E. coli*. ISG15 was incubated with SARS-CoV-2 PLpro (lanes 3–6). SARS-CoV-2 PLpro was present at 4 nM, ISG15 was present at 10 µM. For lane 4 to 6, famotidine was present at 100 µM, 10 µM and 1 µM respectively. Control was without enzyme (lane 2). Proteins were resolved by 15% SDS-PAGE and revealed by Coomassie blue staining. The molecular weights of the marker proteins are indicated on the left of the gel.

Initial testing of famotidine as a medical countermeasure for COVID-19 emerged from a computational molecular docking effort aimed at identifying inhibitors of the papain-like protease (PLpro) of SARS-CoV-2 (Baez-Santos et al., 2015; Daczkowski et al., 2017). In addition to processing the viral polyprotein, the papain-like protease from coronaviruses (PLpro) is known to remove the cellular substrates ubiquitin and the interferon stimulated gene 15 (ISG15) from host cell proteins by cleaving the C-terminal end of the consensus sequence LXGG, a process termed deISGylation (Han et al., 2005; Mielech et al., 2014). Here, we used the enzymatic reaction of SARS-CoV-2 PLpro on ISG15 to assess the potential inhibition of PLpro by famotidine. The cleavage of the 8 C-terminal amino acids of ISG15 by PLpro is clearly detected by SDS-PAGE (Figure 1, lanes 2 and 3). However, the addition of 1–100 µM famotidine to the reaction does not significantly reduce the amount of ISG15 cleaved during the assay (Figure 1, lanes 4–6), thus suggesting that famotidine does not inhibit SARS-CoV-2 PLpro. A previous virtual screening report (Wu C. et al., 2020) suggested that famotidine might bind to the 3 chymotrypsin-like protease (3CLpro), more commonly referred to as the main protease (Mpro), however this mechanism was recently discounted (Anson et al., 2020).

#### Famotidine Does Not Directly Inhibit SARS-CoV-2 Infection

**FIGURE 2:**
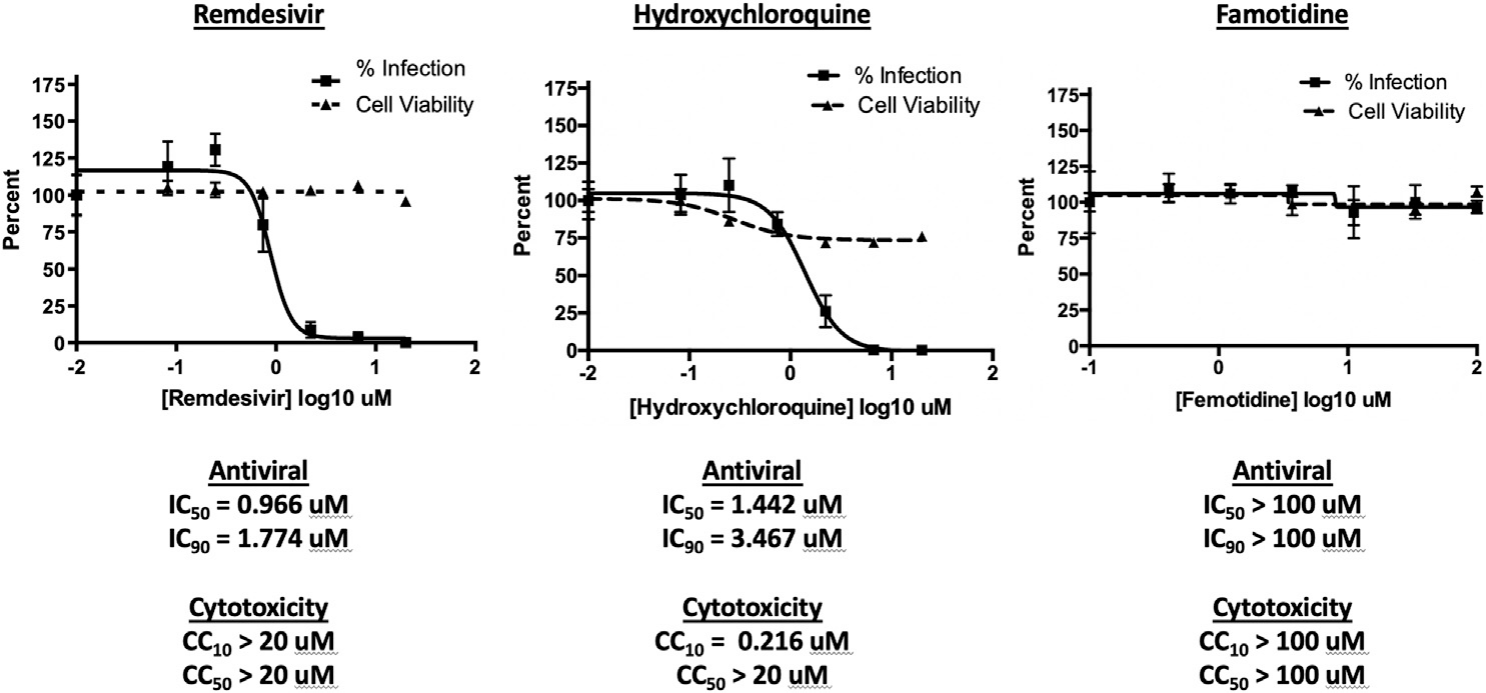
Famotidine does not directly inhibit SARS-CoV-2 infection. To assess the possibility that famotidine may inhibit SARS-CoV-2 infection by other routes, a Vero E6 cell-based assay was performed to compare median tissue culture infectious doses (TCID50/mL) of famotidine, remdesivir, and hydroxychloroquine. Vero E6 cells were cultured and infected as described in methods and scored for presence or absence of infection using the surrogate of an immunohistochemical stain for cell-associated SARS-CoV-2 NP protein as scored by imaging cytometer. Non-specific cytotoxicity (inverse of viability) was assessed using an MTT assay. As appropriate, infected supernatants were assayed for infectious viral titer using the TCID50 method. Results are displayed as % inhibition of the viral infection Vero E6 cells as a function of tested pharmaceutical, % infected cell viability, pharmaceutical agent concentration necessary to achieve 50% or 90% replication inhibition (IC50, IC90 respectively), and pharmaceutical agent concentration required to yield 10% or 50% reduction in cell viability (CC10, CC50).

To assess the possibility that famotidine may inhibit SARS-CoV-2 infection by other routes, a Vero E6 cell-based assay was performed to compare median tissue culture infectious doses (TCID50/mL) of famotidine, remdesivir, and hydroxychloroquine (Figure 2). While both remdesivir and hydroxychloroquine demonstrated antiviral activity, no inhibition of SARS-CoV-2 infection was observed with famotidine.

### Human Receptors

#### Famotidine Does Not Act via Sigma-1 or -2 Receptor Binding

**FIGURE 3:**
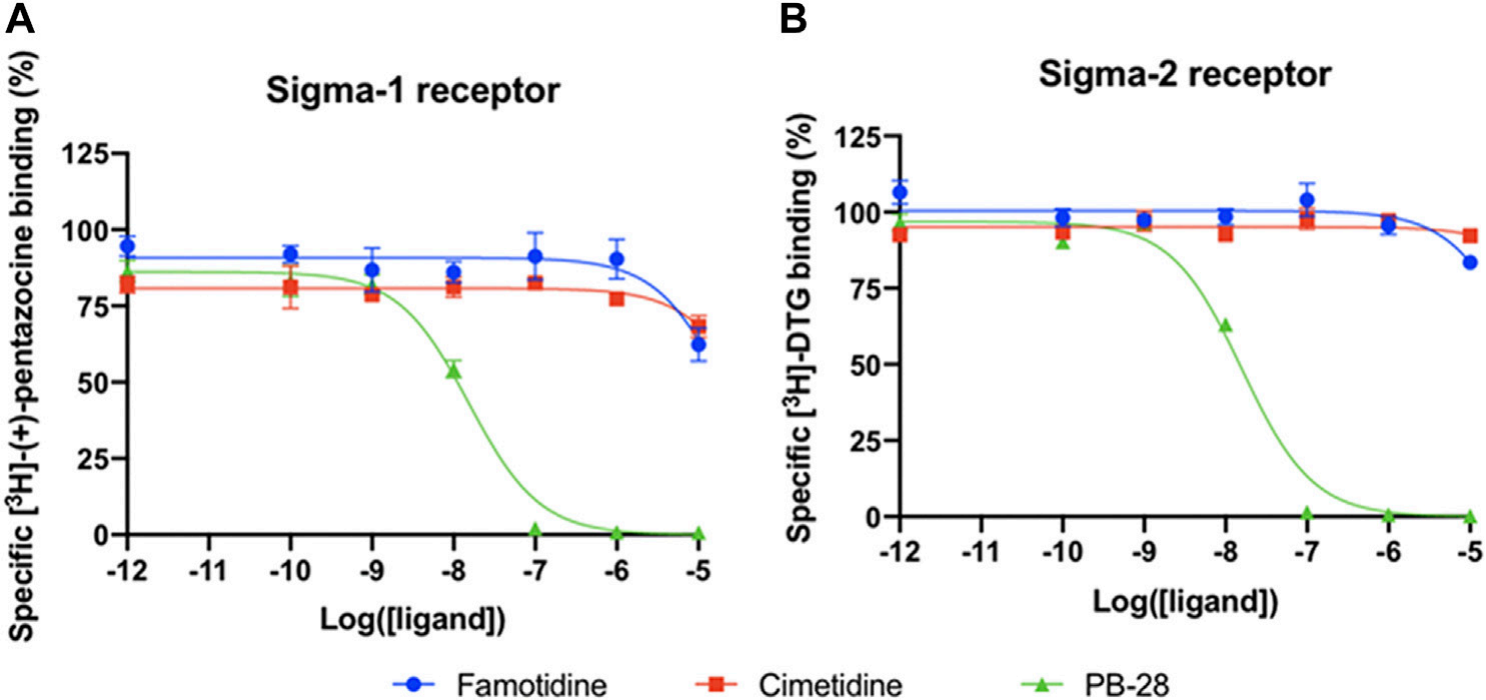
Competition binding curves of Famotidine (blue circles), Cimetidine (red squares), and PB-28 (green triangle), a potent sigma receptor ligand as positive control. **(A)** [3H](+)-pentazocine competition curves in Expi293 membranes expressing sigma-1. **(B)** [3H]DTG competition curves in Expi293 membranes expressing sigma-2 (TMEM97).

A wide-ranging study recently presented a map of interactions between viral and host proteins (Gordon et al., 2020). It was shown that regulation of the sigma-1 and sigma-2 receptors had antiviral effects. Sigma and histamine receptors share several ligands in common, like the antipsychotic haloperidol, the antihistamines astemizole and clemastine, the antidepressive clomipramine, and many more. As such, we tested for possible interaction between famotidine and sigma-1 or sigma-2 receptors (Figure 3). We performed radioligand competition binding experiments using cloned sigma receptors, following established procedures (Schmidt et al., 2016;Alon et al., 2017). In these assays, famotidine showed no detectable displacement of radioligand probes for either sigma-1 or sigma-2 receptors at famotidine concentrations up to 10 μM. Hence, famotidine’s binding to sigma-1 and sigma-2 receptors is likely negligible at physiologically relevant concentrations.

#### Famotidine is Selective for Receptor H_2_

**FIGURE 4:**
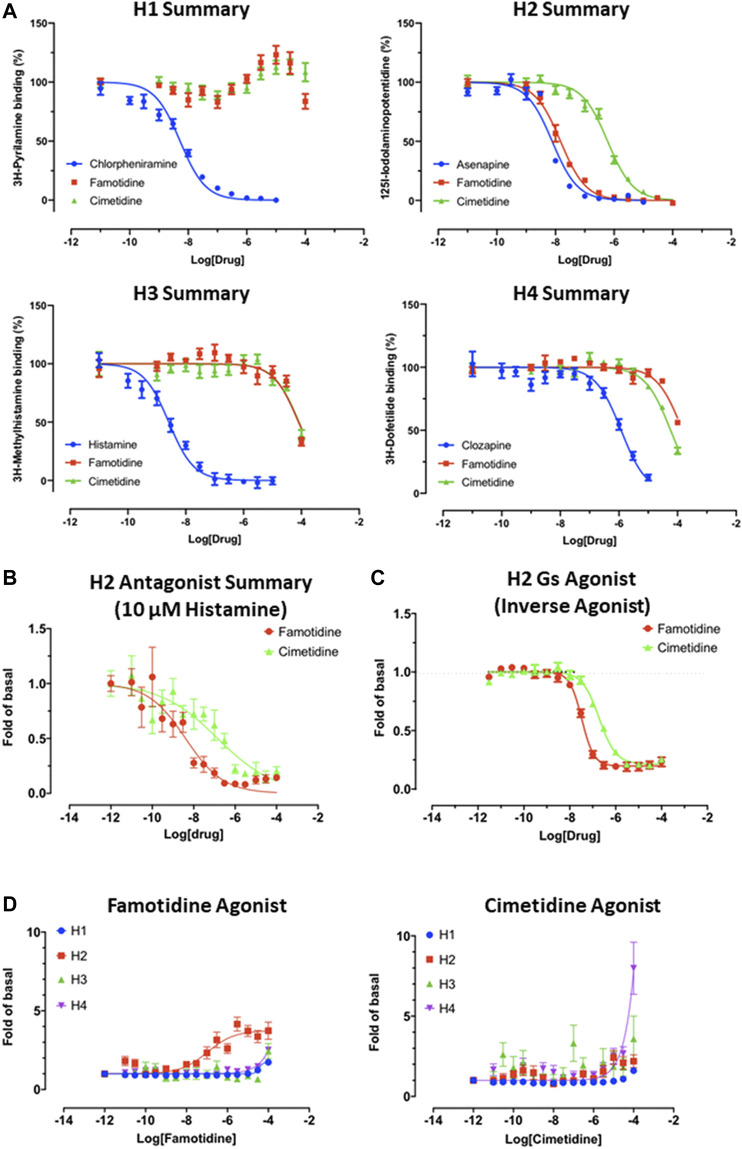
Famotidine and cimetidine activity on histamine receptors. Experiments performed in duplicate. **(A)** Competitive binding dose-response curves for famotidine and cimetidine on four histamine receptors with reference compounds. **(B)** The partial agonist, famotidine, shows antagonist activity of H2 in the presence of potent endogenous agonist, histamine. **(C)** Inverse agonism of famotidine and cimetidine on H2, whereas histamine stimulated cAMP production by 20-fold of basal (*N* = 2). **(D)** Arrestin recruitment by famotidine **(left)** and cimetidine **(right)** upon interaction with histamine receptors.

As is well-known (Bertaccini et al., 1986), famotidine is a selective blocker of the histamine H_2_ receptor with a measured affinity of approximately 14 nM, substantially more active than the affinity (590 nM) of cimetidine for histamine H_2_ receptors (Figure 4A). As shown below, data generated demonstrate that famotidine has highly efficacious inverse agonist activity (reducing basal activity by 75%) with a potency of 33 nM (Figure 4C). Intriguingly, and unlike cimetidine, while famotidine acts to block Gs protein signaling, it actually acts as a partial agonist of arrestin recruitment, with an efficacy of about 15% that of histamine, and an EC50 of 105 nM (Figure 4D), suggesting that the molecule promotes arrestin-scaffolded signaling--such as through the ERK pathway (Alonso et al., 2015), and promotes internalization of the receptor and further non-canonical signaling once internalized (Irannejad and von Zastrow, 2014; Jean-Charles et al., 2017) through an arrestin-biased mechanism. These features distinguish famotidine certainly from cimetidine, and potentially from other H_2_ blockers, as such biased activation of arrestin recruitment for GPCR antagonists, while not unprecedented, is not common.

#### Famotidine May Activate Other GPCRs

**FIGURE 5:**
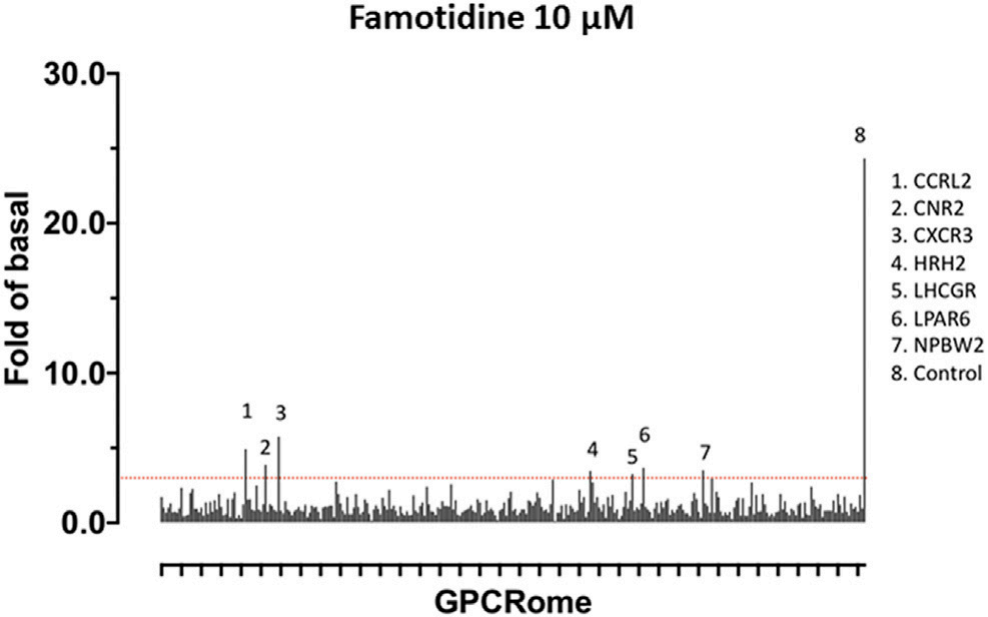
Screen for activation of 318 receptors of the GPCR-ome. To test whether famotidine may act via other G-coupled protein receptors (GPCRs) in addition to its activity as an inverse agonist for the histamine H2 receptor, a screening assay method was applied to detect potential agonist activity of famotidine (10 microM final) when interacting with each of the 318 known human GPCRs. Prior surveys with other pharmaceuticals have defined the baseline for non-specific signal in this assay at 3x greater than the corresponding basal signal. GPCRs meeting the screening criteria of >3x baseline are listed. None of these screening signals were verified in follow up studies, yielding the conclusion that famotidine has no agonist activity for other human GPCRs.

A screen for famotidine activation of 318 receptors of the GPCR-ome was performed and revealed only seven receptors with an average fold of basal increase above 3.0, including H_2_ (Figure 5). In all cases, the quadruplicate replicates were not in agreement. Excluding H_2_, none of the activations were reproducible in follow-up studies.

### Famotidine Reaches Functionally Relevant Systemic Concentrations, Whereas Cimetidine Does Not

We calculated predicted steady state concentrations of famotidine and cimetidine at different doses based on published pharmacokinetic and biodistribution data (Lin et al., 1987; Yeh et al., 1987; Lin, 1991). This modeling demonstrated that the different clinical outcomes exhibited by COVID-19 patients taking famotidine vs. cimetidine could be readily explained by the distinctive pharmacokinetic and pharmacodistribution properties of the two agents.

Therapeutic efficacy of a pharmacological antagonist requires that it achieves a steady-state concentration that substantially exceeds the half maximal inhibitory concentration (IC_50_) for its target. Thus, to evaluate the relative systemic effects of famotidine and cimetidine, the IC_50_ values of each agent for the H_2_ receptor were compared to the steady-state plasma concentrations (Css) predicted at standard clinical doses. As demonstrated above, famotidine binds to H_2_ with a concentration of inhibitor at which (under saturating substrate conditions) the reaction rate is half of the maximum reaction rate Vmax (ergo Ki) of 14 nM, whereas cimetidine binds to H_2_ with Ki 586 nM. Previous reports suggest functional IC_50_s are approximately 3x higher, and these data were used for the current analyses (Lin et al., 1987; Lin, 1991). In these reports, the total concentration of inhibitor required to reach 50% inhibition (IC50) for the H_2_ receptor were reported as 13 μg/L (0.039 μM) for famotidine and 400–780 μg/L (1.59–3.09 μM) for cimetidine. Steady state concentration (Css) values were calculated using pharmacokinetic data for dosing, clearance, bioavailability, and volume of distribution as summarized previously (Lin, 1991). Table 1 lists the Css values for both famotidine and cimetidine.

**TABLE 1:** Steady-state concentrations (Css) of Famotidine and Cimetidine at standard doses compared to the half maximal inhibitory concentration (IC50) value of Famotidine or Cimetidine for histamine H_2_ receptor antagonism.

IC50 or Css	Concentration (mass/volume)	Concentration (molarity)		
Famotidine				
IC50 Histamine H_2_	13 μg/l	0.039 μM		
IC50 Neutrophil H_2_ O_2_- assay	67 μg/l	0.201 μM		
IC50 Neutrophil H_2_ cAMP assay	8 μg/l	0.024 μM		
IC50 Eosinophil H_2_ cAMP assay	53.6 μg/l	0.158 μM		

IC50 Mast Cell H_2_ cAMP increase	Not determined			
	Total Concentration (mass/volume)	Total Concentration (mass/volume)	Free drug Concentration^c^ (mass/volume)	Free drug Concentration^c^ (molarity)
Css (20 mg tablet p.o. q.d.)	17.7 μg/	0.053 μM	14.2 μg/l	0.042 μM
Css (20 mg capsule p.o. q.d.)	18.4 μg/l	0.055 μM	14.7 μg/l	0.044 μM
Css (20 mg tablet p.o. b.i.d.)	35.4 μg/l	0.105 μM	28.3 μg/l	0.084 μM
Css (20 mg capsule p.o. b.i.d.)	36.8 μg/l	0.109 μM	29.4 μg/l	0.087 μM
Css (20 mg tablet p.o. t.i.d.)	53.1 μg/l	0.157 μM	42.5 μg/l	0.126 μM
Css (20 mg capsule p.o. t.i.d.)	55.3 μg/l	0.164 μM	44.2 μg/l	0.131 μM
Css (40 mg tablet p.o. t.i.d.)^a^	55.4 μg/l	0.164 μM	44.3 μg/l	0.131 μM
Css (40 mg tablet p.o. t.i.d.)^b^	80.8 μg/l	0.239 μM	64.6 μg/l	0.192 μM
Css (60 mg tablet p.o. t.i.d.)	144.3 μg/l	0.425 μM	115.4 μg/l	0.340 μM
Css (120 mg IV every 8 h)	1,290 μg/l	1.092 μM	1,032 μg/l	0.874 μM
Cimetidine				
IC50 Histamine H2	400–780 μg/l	1.59–3.09 μM		
Css (200 mg tablet p.o. q.d.)	175 μg/l	0.69 μM	140 μg/l	0.055 μM
Css (300 mg tablet p.o. q.d.)	226 μg/l	0.90 μM	180.1 μg/l	0.720 μM

^a^ Calculated using pK data reported by Lin et al. (1987).

^b^ Calculated using pK data reported by Yeh et al. (1987).

^c^ Both famotidine and cimetidine are approximately 20% protein bound in systemic circulation (Somogyi and Gugler, 1983; Echizen and Ishizaki, 1991).

In primary human neutrophils and eosinophils, H_2_ activation by histamine inhibits neutrophil effector functions including O_2_- release (Gespach and Abita, 1982; Burde et al., 1989), platelet-activating-factor induced chemotaxis (Rabier et al., 1989) and leukotriene biosynthesis (Flamand et al., 2004). Eosinophil functions are also inhibited by H_2_ activation; histamine binding diminishes eosinophil peroxidase release (Ezeamuzie and Philips, 2000) and, at high concentrations, inhibits eosinophil chemotaxis (Clark et al., 1975; Wadee et al., 1980). Famotidine is one of the most effective antagonists of these H_2_-mediated histamine effects on neutrophils and eosinophils (Reher et al., 2012). IC_50_ for two measures that relate to these phenotypes are also listed in Table 1. Mast cells express histamine H_2_ and H_4_ receptors, and histamine-induced increase of cAMP in mast cells is inhibited by famotidine (Lippert et al., 2004). 10 μM famotidine pre-incubation blocks histamine-induced cAMP increase in human skin mast cells, however, the IC_50_ for this effect has not been determined (Lippert et al., 2004).

At all dosing regimens, the Css for famotidine exceeds the general IC_50_ value for the H_2_ receptor, and at the twice daily (b.i.d.) and thrice daily (t.i.d.) dosing of 20 and 40 mg, the Css for unbound famotidine is 2-5 fold greater than the H_2_ IC_50_. Also calculated and summarized is the Css for the intravenous dosage currently being administered in clinical trial NCT04370262 and that dose exceeds famotidine IC_50_ by greater than 20-fold. In contrast, unbound cimetidine levels at standard doses of 200 or 300 mg daily (q.d.), achieve a Css that is a fraction of the reported IC50 range of 400–780 μg/l.

### Comparative Analysis of Mast Cell Density in Lung Parenchyma of SARS-CoV-2 Infected African Green Macaques

**FIGURE 6:**
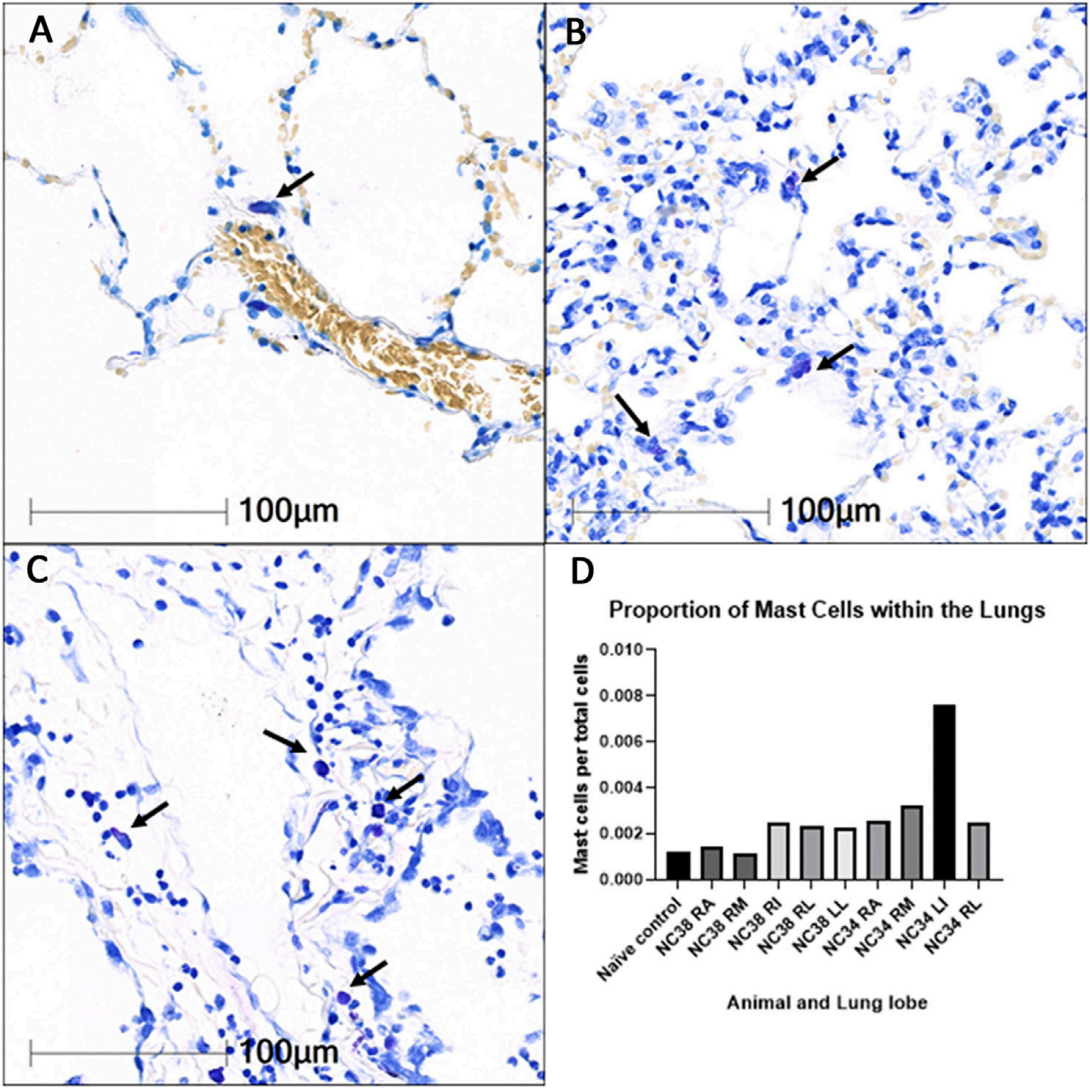
Infiltration of mast cells into the pulmonary parenchyma of SARS-CoV-2 infected African Green monkeys (AGMs). 20× magnification. Toluidine blue stain. RA: right anterior; RM: right middle; RI: right intermediate; RL: right lower; LL: left lower.

Images and data summarizing studies of infiltration of mast cells into the pulmonary parenchyma of SARS-CoV-2 African Green monkeys (AGMs) are summarized in Figure 6. As shown in Panel A (20x magnification), in naïve AGM lung parenchyma, mast cells (arrow) are rarely observed within alveolar interstitium. Panels B and C (20x), increased numbers of mast cells (arrows) are seen within the interstitium of SARS-CoV-2 infected AGMs that both developed mild (B) and severe pneumonia (C). Panel D summarizes the proportion of total cells that are mast cells is elevated in all lobes of SARS-CoV-2 infected AGMs in comparison to naïve control tissue.

### Case History, Severe COVID-19 Outpatient Treatment With Famotidine

Patient JM is a 47 year old male who received PCR diagnosis of COVID-19 after 8 days of complaints of diarrhea, abdominal cramping, eructation, low energy, dry cough, arthralgia, myalgia, anosmia and ageusia. The patient has a history of hypertension (10 years), Type II diabetes (4 years), hypercholesterolemia (3 years) and gout (10 years). Current medications included Metformin, Allopurinol, Lisinopril, and Atorvastatin. He is employed as a hospital maintenance worker in the hospital to which he presented.

Contact tracing revealed that his son (same household) had developed COVID-19 symptoms 12 days prior. Receipt on day 8 of positive PCR diagnosis (from a prior outpatient intranasal swab sample) coincided with onset of fever (102oF), night sweats, shortness of breath and a feeling of chest pressure. Famotidine (“PEPCID AC ®” 60 mg p.o. t.i.d. = 2.24 mg/ft2 t.i.d) was initiated upon receiving the PCR diagnosis due to symptoms meeting FDA criteria for severe COVID-19, combined with high-risk pre-existing conditions. The famotidine drug regime was continued for 30 days. After initiating famotidine in the evening, the patient was able to sleep through the night and reported complete relief from the chest pressure sensation, reduction in cough, but continued to be febrile (101.6°F).

**FIGURE 7:**
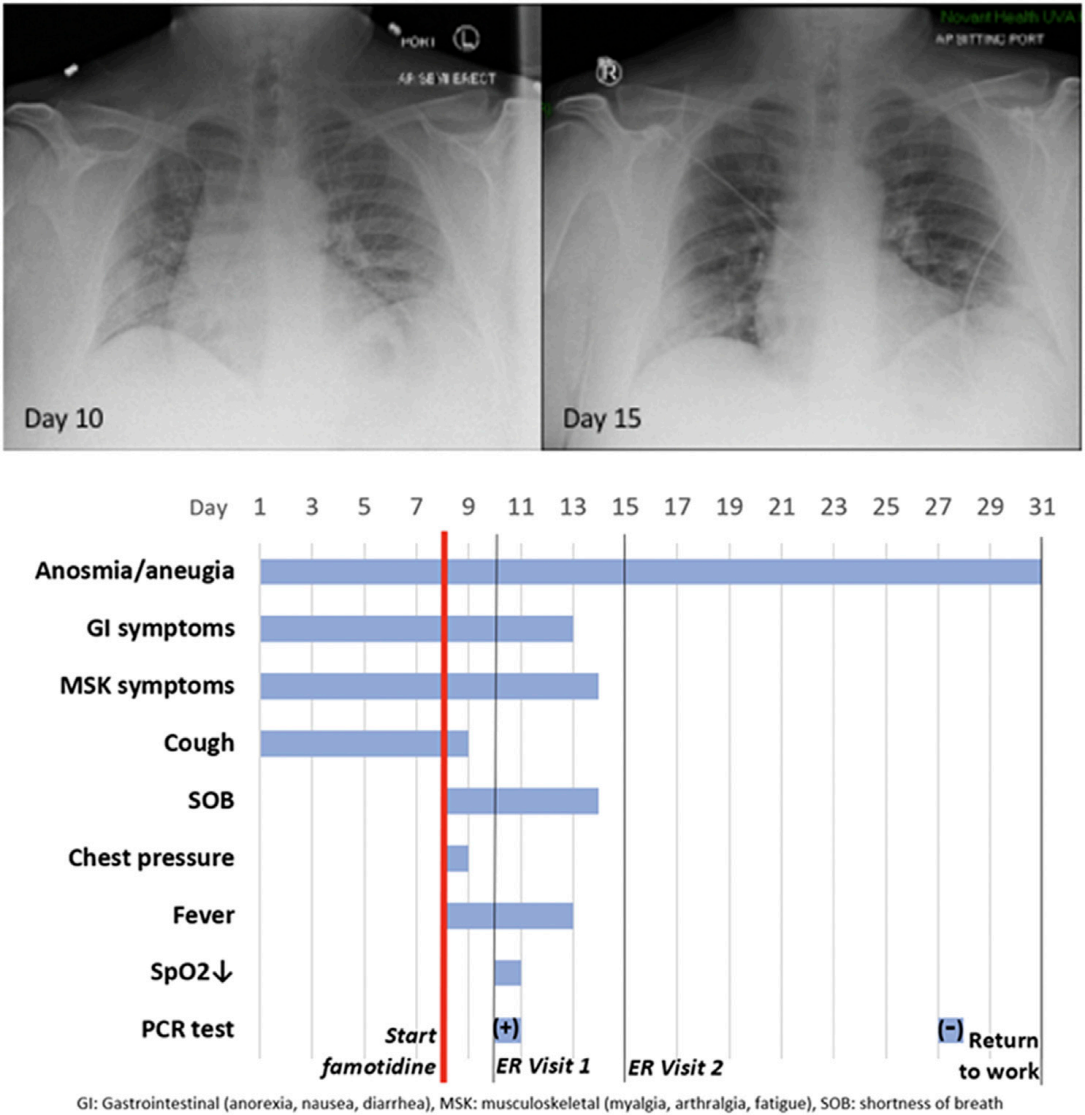
ase Study JM: CXR and Timeline. Famotidine (60 mg PO tid) was started on Day 8 from start of symptoms. It was continued for 30 days. The anosmia and ageusia are still present at Day 50.

On day 10, he presented to the emergency room (ER) with continuing complaints of diarrhea, abdominal cramping, eructation, low energy, dry cough, arthralgia, myalgia, anosmia and ageusia and shortness of breath on exertion. Day 10ER physical examination, including the chest, was unremarkable and vital signs were normal. The patient body mass index (BMI) was 36 (Du Bois BSA 26.78 ft^2^). SpO2 was 93%, rising to 97 and 99% on 3 L/min by nasal cannula over the next 30 min. An intranasal sample was obtained for SARS-CoV-2 rtPCR diagnostic analysis. Comprehensive metabolic panel showed a mild decrease in serum sodium and chloride with hyperglycemia (260 mg/dl). Complete blood count (CBC) was normal, specifically including the lymphocyte count. Urinalysis showed a specific gravity of 1.025 but was otherwise normal. A portable chest X-ray had poor inspiration but was interpreted as showing “bibasilar areas of airspace disease” consistent with COVID-19 (Figure 7, chest x-ray [CXR] day 10). The patient was diagnosed as dehydrated, given ondansetron IV, 1 L IV of normal saline and discharged home with a hospital pulse oximeter. At the time of departure, he had an SpO2 of 94% on room air that did not drop with ambulation.

The patient again presented to the emergency room on day 15 after experiencing near-syncope during showering. Physical examination was unremarkable. Vital signs were normal. SpO2 showed values of 98, 93, and 97% on room air over the 2 h period. Basic metabolic panel showed only hyperglycemia (266 mg/dl). CBC was normal except for a mild lymphopenia (0.96; reference range 1.00–3.00 × 103/μL) and mild monocytosis (0.87; 0.20–0.80 × 103/μL). Chest X-ray was interpreted as showing “Faint patchy consolidation of lung bases bilaterally, similar to perhaps minimally improved at the lower left lung base compared to prior” (Figure 6 CXR day 15). The patient was placed on azithromycin and discharged to home.

On days 27 and 28 after initial symptoms, he tested negative (2x, successive days) for SARS-CoV-2 nucleic acid by PCR (intranasal swab) and returned to his work at the local hospital 31 days after initial symptoms. 47 days after first developing COVID-19 symptoms he continues to note a lack of ability to taste or smell, but otherwise considers himself largely recovered from COVID-19 (Figure 7 timeline).

Use of famotidine in this patient was recommended due to meeting FDA criteria for severe COVID-19 and his COVID-19 risk factors: male, 47yo, hypertension, obesity (Divoux et al., 2012) and diabetes mellitus Type 2. Although this is an anecdotal example, the patient experienced relief of symptoms overnight upon initiating use of famotidine. While not sufficient to demonstrate proof of cause and effect, this case does provide context for typical COVID-19 presentation and symptoms, as well as support for additional well-controlled famotidine therapeutic clinical trials in an outpatient setting.

## Discussion

Famotidine is an off-patent drug available as either branded (“PEPCID®”) or generic medicines in tablet, capsule or intravenous forms. The general pharmacology of famotidine is well-characterized, with an excellent absorption, distribution, metabolism, excretion and toxicology profile (FDA, 1986). Famotidine is unique among the drugs currently being tested for treatment of COVID-19, in that it is an H_2_ receptor antagonist (and inverse agonist). Famotidine is currently being tested for treating COVID-19 in a double blind randomized clinical trial at high intravenous doses in combination with either hydroxychloroquine or remdesivir (ClinicalTrials.gov Identifier: NCT04370262). A retrospective cohort study of 1,620 hospitalized COVID-19 patients indicates that 84 propensity score matched patients receiving famotidine during hospitalization (oral or IV, 20 mg or 40 mg daily) had a statistically significant reduced risk for death or intubation (adjusted hazard ratio (aHR) 0.42, 95% CI 0.21–0.85) and also a reduced risk for death alone (aHR 0.30, 95% CI 0.11–0.80) (Freedberg et al., 2020). Subsequent famotidine/COVID-19 retrospective data analysis reports have been less clear, ranging from reporting no effect (Cheung et al., 2020; Shoaibi et al., 2020; Yeramaneni et al., 2020) to various levels of protective effect (Mather et al., 2020). To the extent that these retrospective studies report famotidine dosage levels, in all cases the dosages are either unreported (Cheung et al., 2020) or predominantly standard dosing for relief of gastro-esophageal reflux disease (GERD). No retrospective studies involving high dose famotidine (60–80 mg oral 3–4 times per day) have been reported to date. Anecdotal reports and undisclosed data indicating that famotidine provided protection from COVID-19 mortality while neither cimetidine nor proton pump inhibitors were similarly protective lead to an initial inference that the beneficial effects of famotidine were not related to the known on-target activity of the drug (Borrell, 2020). Studies detailed in this report and others, however, indicate that famotidine does not act by directly inhibiting either of the principal SARS-CoV-2 proteases (PLpro or Mpro) (Anson et al., 2020). Vero E6-based cell assays also indicate that famotidine has no direct antiviral activity in this cell line, although antiviral activity in cells that express H_2_ has not been tested. Additional hypotheses that famotidine may act via binding either the sigma-1 or -2 receptors have not been supported by the studies summarized herein.

The most straightforward explanation of the apparent famotidine activity as a COVID-19 therapy is that the drug acts via its antagonism or inverse-agonism of histamine signaling and via its arrestin biased activation—all a result of famotidine binding to histamine receptor H_2_. If true, then it is reasonable to infer that a SARS-CoV-2 infection that results in COVID-19 is at least partially mediated by pathologic histamine release. The anecdotal lack of protection provided by oral administration of the H_2_ antagonist cimetidine can be accounted for by insufficient systemic drug levels after oral administration and does not contradict potential benefit provided by famotidine H_2_ binding. Intravenous cimetidine at sufficient doses may achieve levels high enough for clinical benefit and would further support this hypothesis. Failure to achieve clinical COVID-19 responses with cimetidine may indicate that inverse agonism or other GPCR-mediated effects of famotidine may play an important role in the (preliminary) observed clinical benefits. Analysis of famotidine activity in histamine receptor competition assays indicate that, over the range of clinical steady state famotidine drug levels being tested, famotidine is specific for H_2_. Therefore, off-target antagonism of histamine H_1_ receptor, H_3_ receptor, or H_4_ receptor is unlikely to contribute to famotidine-mediated effects.

Steady state famotidine concentrations sufficient to elicit H_2_ antagonism (and inverse agonism) are readily achieved using inexpensive oral tablets and safe dosage levels. As summarized above, the famotidine dosage employed in the retrospective hospital studies currently available which examine famotidine effects on COVID-19 outcomes appears to be levels (20–40 mg daily) which are unlikely to fully inhibit histamine-mediated systemic effects at the H_2_ receptor (Freedberg et al., 2020). In contrast, study NCT04370262 administers intravascular famotidine doses that are more than 20-fold greater than the IC_50_ for antagonism of H_2_. Other prospective randomized clinical studies involving high dose famotidine for treating COVID-19 are in progress [Samimagham et al. (2020), NCT NCT04504240]. The data presented herein provides a rationale for famotidine dose selection to maintain a steady state concentration at a reasonable multiple of the IC_50_ for systemic antagonism of H_2_ and indicate that oral tablet dosages of between 40 mg every 8 h to 80 mg every 8 h should be sufficient to insure maximal H_2_ target effects. As famotidine is primarily cleared by the kidney, adequate renal function is required for higher dosages (FDA, 1986).

In addition to H_2_ antagonism, famotidine may also act as an inverse agonist thereby lowering the concentration of cyclic-Adenosine Monophosphate (c-AMP) (Alonso et al., 2015). Endothelial cell permeability has been attributed to histamine H_2_ activation and is blunted by famotidine pretreatment (Luo et al., 2013). Histamine, bradykinin and des-arg-bradykinin receptor engagements can lead to increased endothelial permeability through a common pathway that results in AKT-1 activation (Di Lorenzo et al., 2009). The H_2_ receptor also signals through Gq/11 proteins, resulting in inositol phosphate formation and increases in cytosolic Ca^2+^ concentrations which may account for the increased endothelial cell fluid permeability (Panula et al., 2015).

One alternative hypothesis is that famotidine may not only inhibit signaling through the H_2_ receptor but may also engage in cross talk with the kinin B1 receptor, which moderates the response of endothelial cells to DABK and DAKD ligands. Data provided here in are not consistent with this hypothesis; no activation of bradykinin receptor B1 or B2 were observed in quadruplicate replicate TANGO assay.

Another alternative hypothesis is that famotidine is active in mitigating the effects of neutrophil sensitivity to activation yielding extracellular traps (Radermecker et al., 2020). At autopsy, the microvascular thrombi of COVID-19 demonstrate large number of neutrophils, and it has now been shown that overshooting, global neutrophil activation is present in severe and critical.

COVID-19 (Nicolai et al., 2020; Radermecker et al., 2020). Hyperactivated neutrophils and enhanced neutrophil extracellular traps (NETS) function in the NET-like structures subsequently identified in pulmonary, kidney, and heart specimens. Reactive Oxygen species are important for NETosis, and these effects are at least partially mitigated by histamine H_2_ blockers including famotidine and cimetidine (Mikawa et al., 1999). Previous pharmacological studies of famotidine have shown a dose dependent attenuation of intracellular calcium concentrations in neutrophils and at high doses that reactive oxygen species are attenuated [see Tomera and Kittah (2020b) for further details]. This would explain the clinical observations that elevated D-dimers and pulmonary emboli become responsive to standard anticoagulation therapy when given HD famotidine (Tomera and Kittah, 2020a; Radermecker et al., 2020).

While COVID-19 symptoms affect multiple organ systems, respiratory failure due to acute respiratory distress syndrome (ARDS) is the most common cause of death. Examination of RNA expression profiles of the cells which contribute to lung anatomy and function demonstrate the presence of multiple ACE2/TMPRSS2 positive cell types susceptible to SARS-CoV-2 infection in the lung. In addition, these and other associated lung cells that are positive for histamine receptors H_1_ and H_2_ could respond to local histamine release following mast cell degranulation (Krystel-Whittemore et al., 2015), and therefore those cells positive for H_2_ may be responsive to famotidine effects.

**FIGURE 8:**
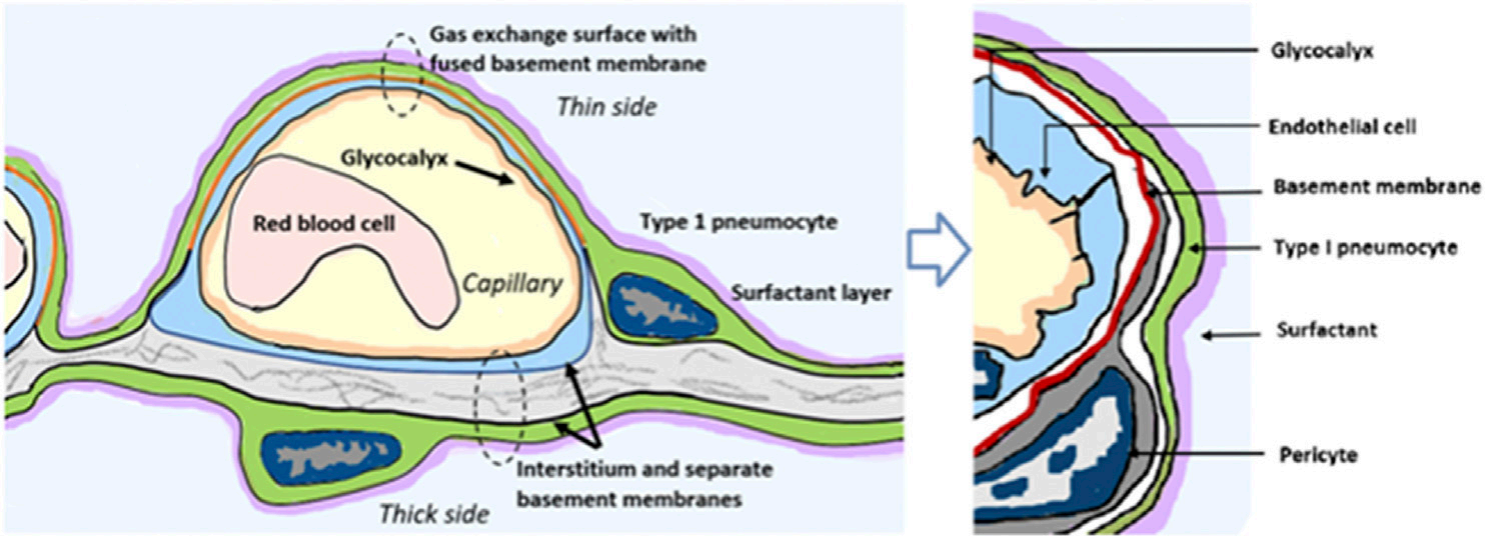
Lung alveolus cell interactions and gas exchange. Schematic diagram illustrating relevant cellular and tissue microanatomy of the pulmonary alveolus. Pulmonary edema results from loss of a regulation of fluid transfer that occurs at several levels in the alveolus, including disrupted capillary wall components, surfactant, Type I and II pneumocytes, as well as the pulmonary pericytes which are a histamine-responsive contractile cell which both synthesize the endothelial basement membrane and regulate blood flow in the precapillary arteriole, the capillary and the postcapillary venule via contraction and relaxation response to histamine and other signaling molecules.

To understand how famotidine may act to reduce pulmonary COVID-19 symptoms requires an understanding of COVID-19 lung pathophysiology, which appears to have two principal disease phases. In turn, this requires an appreciation of pulmonary tissue and cell types. Pulmonary edema results from loss of a regulation of fluid transfer that occurs at several levels in the alveolus, as diagrammed in Figure 8. In the capillary wall, there are the glycocalyx, the endothelial cell with associated tight junctions, and the basement membrane. In the epithelium there is a surfactant layer on the alveolar lining fluid, manufactured and secreted by the Type II pneumocyte, and the Type I pneumocyte itself with its tight junctions and negatively charged basement membrane which restricts albumin. The pulmonary pericytes located in the terminal conducting airway region play a critical role in synthesizing the endothelial basement membrane and regulating blood flow in the precapillary arteriole, the capillary and the postcapillary venule. Disruption of any of these cells or layers can lead to edema. This edema fluid may be a transudate in milder dysfunctions or an exudate when inflammation or necrosis develop. Two possible pathologies that could result in edema of the alveolar wall and space include infection of cells by SARS-CoV-2 and mast cell degranulation with release of hundreds of compounds that can impact on cellular and basement membrane functions, glycocalyx and tight junction integrity. These compounds include histamine, bradykinin, heparin, tryptase and cytokines.

**FIGURE 9:**
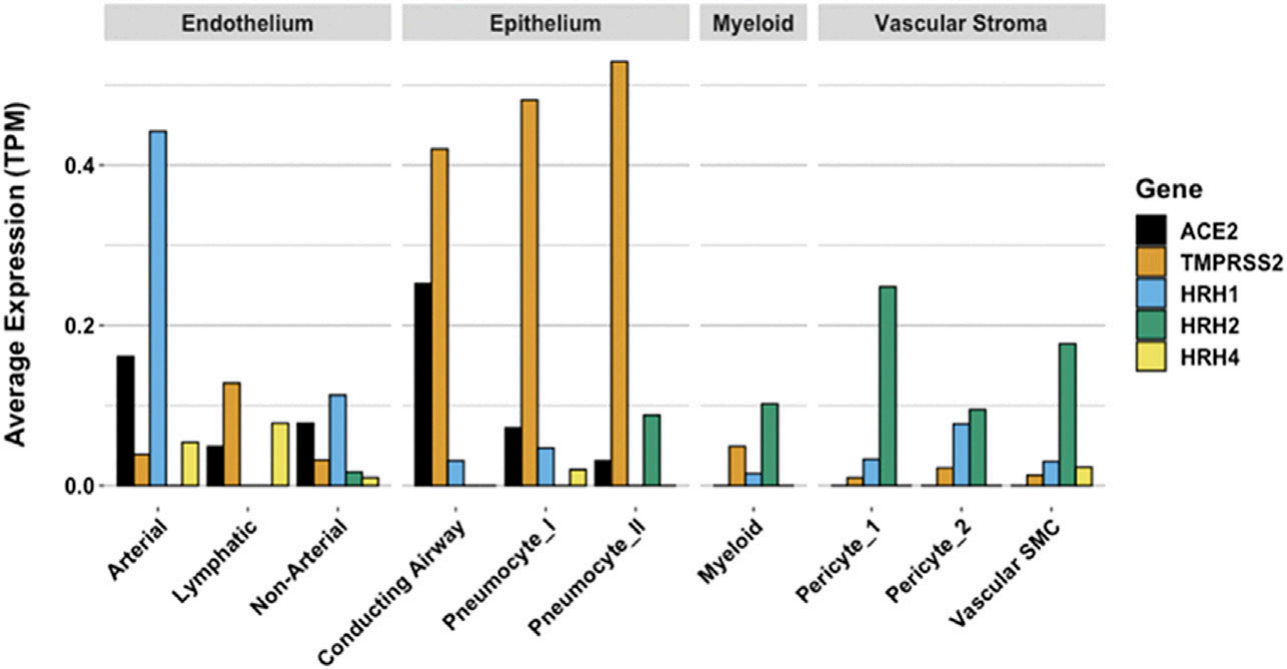
Human single cell lung gene expression normalized to transcripts per million (TPM) from LunGENS web portal (Du et al., 2015). Single cell lung gene expression patterns from the Dropseq PND1 experiment for angiotensin-converting enzyme 2 (ACE2: black), transmembrane protease, serine 2 (TMPRSS2; orange), and histamine receptors H1 (blue), H2 (green), and H4 (yellow).

Gene expression patterns of these pulmonary cells provide insight into which cells are likely to be infected, and which express the H_2_ receptor that could be directly impacted by famotidine treatment and resulting H_2_ antagonism or inverse agonism (Figure 9). These patterns suggest that epithelial cells and endothelial cells are more likely to be infected based on ACE2 and TMPRSS2 expression patterns in those cell types. The cells most likely to show a famotidine effect include Type 2 pneumocytes, smooth muscle cells, pericytes, and myeloid granulocytes (which includes mast cells, neutrophils and eosinophils).

The limited tissue pathology available from early COVID-19 cases seems to support both viral infection as well as histamine effects in the lung. In a singular study of early COVID-19, Sufang Tian et al. (2020b) describe the viral lung pathology of early COVID-19 in tissue resected for cancer. Their photomicrographs show two different patterns of disease. As shown in Figure 9 panel B, some samples of this lung tissue demonstrate the usual mononuclear inflammatory pattern of interstitial pneumonitis and fibrinous exudate that one would associate with a viral infection. It is striking that no neutrophils or eosinophils are observed in the inflammatory infiltrate. One explanation is that H2 activation of neutrophils inhibits neutrophil effector functions including O_2_- release (Gespach and Abita, 1982; Burde et al., 1989), platelet-activating-factor induced chemotaxis (Rabier et al., 1989) and leukotriene biosynthesis (Flamand et al., 2004). Eosinophil functions are also inhibited by H_2_ activation; histamine binding diminishes eosinophil peroxidase release (Ezeamuzie and Philips, 2000) and, at high concentrations, inhibits eosinophil chemotaxis (Clark et al., 1975; Wadee et al., 1980).

**FIGURE 10:**
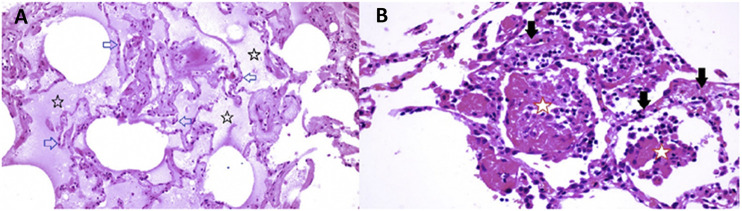
Lung pathology of early COVID-19. Early COVID-19 pulmonary histopathology, illustrating an atypical viral pathology pattern of interstitial and alveolar edema together with alveolar septae which retain normal architecture. Atypical for viral pneumonia, this resection from early in the course of COVID-19 disease lacks inflammation, and the accumulated fluid appears to be a transudate.

The reports of Tian et al. (2020b) and Zeng et al. (2020) also include images in which there is interstitial and alveolar edema while the alveolar septae retain normal architecture (Figure 10 panel A). This is not a pattern typically observed in viral infection, as there is no inflammation, and the fluid appears to be a transudate. It is consistent with dysregulation of the fluid barrier due to the effect of histamine or other mast cell products on endothelial cells, pericytes or Type II pneumocytes. Increased endothelial permeability due to histamine is driven by H1 receptor activation, and so if any potential famotidine treatment effect on these cells occurs it would most likely be indirect by inhibition of mast cell degranulation. Forskolin activates the enzyme adenylyl cyclase and increases intracellular levels of cAMP, and can be used to inhibit the release of histamine from human basophils and mast cells (Marone et al., 1986). Histamine may act as an autocrine regulator of mast cell cytokine and TNF-a release in a PGE2-dependent fashion. Based on *in vitro* studies, this autocrine feedback appears to be mediated by H_2_ and H_3_. Endothelial cells are also susceptible to infection by SARS-CoV-2. Mast cell degranulation-related pulmonary edema could correlate with the early phase silent hypoxia and the high compliance non-ARDS ventilation pattern associated with shortness of breath (Couzin-Frankel, 2020). The image in Figure 10 panel B does not permit evaluation for microvascular thrombi.

Eighty four year old female undergoing right middle lobe (RML) resection for adenocarcinoma. On Day 6 of hospitalization a CT scan showed a ground glass opacity (GGO) in the RML in addition to the tumor mass. Lobectomy was performed on Day 12. On Day 13 (Day 1 post-operation), CT scan showed bilateral bibasilar GGO. On Day 16, she developed typical COVID-19 symptoms with cough, dyspnea and chest tightness. Capillary O_2_ saturation ranged from 77–88%. Death ensued on Day 29. SARS-CoV-2 was confirmed by nasal swab (Tian et al., 2020b)

Panel A (RML): There is extensive pulmonary edema consistent with a transudate (open black stars). Alveolar septae appear normal and there is no inflammation (open blue arrows). Features are not suggestive of an infection.

Panel B (RML): There is fibrinous exudate in the alveolar spaces (open red stars). Alveolar septae show edema and a mononuclear infiltrate (solid black arrows). No neutrophils are identified. There is no significant diffuse alveolar damage of ARDS. Features are typical of an interstitial viral pneumonia.

These findings are supported in a separate autopsy case report of a patient dying 5 days after onset of COVID-19 symptoms. In this case, photomicrographs also show a non-inflammatory transudative-type edema (Schweitzer et al., 2020). In both of these studies, the observed non-inflammatory edema in early stage COVID-19 pulmonary disease is consistent with histamine release by mast cells.

Mast cell degranulation correlates with the COVID-19 natural history that progresses through functionally and clinically different early and later phases. Most SARS-CoV-2 infections follow the typical early phase pattern of any lower respiratory virus, in which a majority of patients have asymptomatic or minimal disease, while a minority go on to later phase acute respiratory distress syndrome (ARDS). Within this spectrum typical of any severe viral disease, COVID-19 has a number of distinctive features. In the out-patient setting, early COVID-19 is usually indistinguishable from other “influenza-like illnesses”, presenting with various non-specific symptoms ranging from sore throat, headache and diarrhea to fever, cough, and myalgias. In these first few days however, COVID-2 may also be associated with anosmia, a unique feature (Eliezer et al., 2020). It is toward the end of the first week of symptoms that COVID-19 patients develop shortness of breath (SOB). This follows cough and fever by several days, a feature not typical of other viruses (Cohen et al., 2020). On physical examination of COVID-19 patients with SOB, the oxygen saturation drops dramatically on exertion. CT scan will usually show bilateral bibasilar ground glass opacifications consistent with pulmonary edema. Nasopharyngeal swabs test positive for SARS-CoV-19. This SOB correlates with a distinctive clinical phenotype of hypoxia with near normal compliance (i.e., >50 mLcmH_2_O). Some authors attribute this to a loss of pulmonary vasoconstriction, one cause of which could be histamine effect on the H_2_ receptors of pericytes and/or vascular smooth muscle. H_1_-related edema and microthrombosis of lung vessels could also be causes. These are the patients that PEEP ventilation will not help, as there are no recruitable alveoli. These patients are helped by lying prone (Gattinoni et al., 2020). It is at this stage that the patient is at greatest risk to progress onto the serious complications of later disease, especially ARDS with its 60–80% mortality if ventilation is required. Patients may also present with additional neurological symptoms and complications including ischemic stroke (Filatov et al., 2020; Mao et al., 2020; Qureshi et al., 2020). Cardiac complications of later COVID-19 include myocarditis, acute myocardial infarction, heart failure, dysrhythmias, and venous thromboembolic events (Long et al., 2020; Mahmud et al., 2020).

**FIGURE 11:**
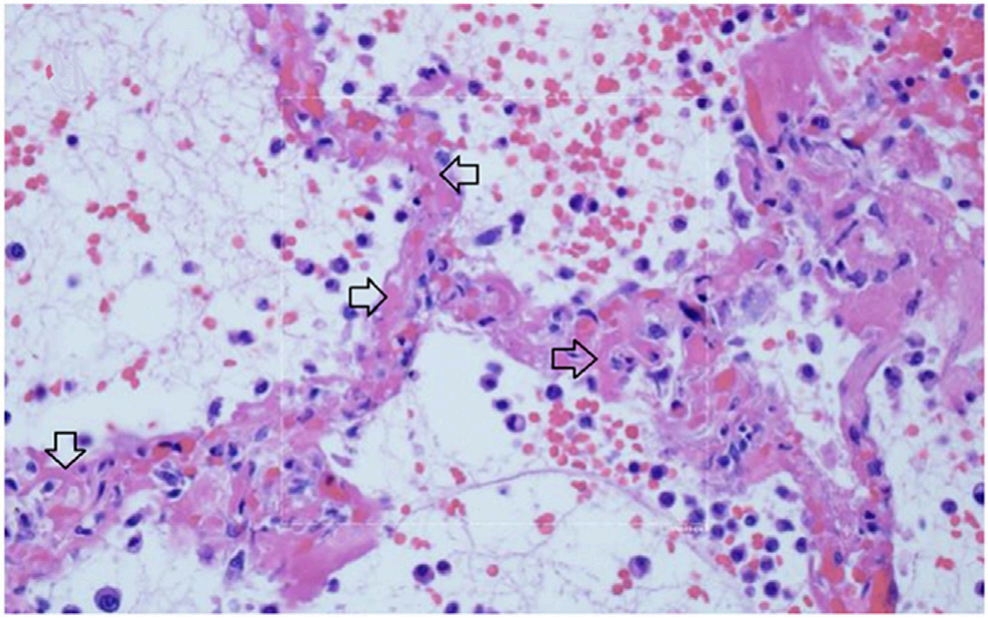
Micro-thrombosis in the pulmonary microvasculature in COVID-19 at autopsy (Magro et al., 2020). There is widening of the alveolar septae by extensive fibrinous occlusion of capillaries (open black arrows). There is alveolar space edema with red blood cell extravasation. Septae show a mild mononuclear infiltrate. Alveolar edema shows neutrophils in proportion to the blood.

Multiple studies have demonstrated a hypercoagulable state in COVID-19 patients requiring hospitalization. Results from a recent large autopsy study suggests that there is also a novel lung-centric coagulopathy that manifests as a small vessel microthrombosis. Based on this study, there are indications that over 50% of patients dying of COVID-19 have pulmonary microthrombosis (Carsana et al., 2020). This thrombosis is not only in arterial vessels, but also can be found in alveolar capillaries in the absence of inflammation and ARDS, as seen in Figure 11 (Magro et al., 2020).

Capillary wall disruption accompanied by fibrin deposition and red cell extravasation, with neutrophils in the septa and within the alveolar spaces (Hematoxylin and eosin, 1000×). For further discussion of microvascular coagulation associated with COVID-19, see (Magro et al., 2020).

Because small microthrombi are difficult to identify on CT scan even with iodinated contrast (Oudkerk et al., 2020), pre-mortem diagnosis is difficult. Laboratory coagulation tests have typically shown normal or mildly prolonged Prothrombin time (PT) and activated partial thromboplastin time (aPTT), normal to increased or slightly decreased platelet counts, elevated fibrinogen levels and very elevated D-dimers (Panigada et al., 2020). Although referred to by some authors as a DIC-like state, this pulmonary microthrombosis does not appear as a typical coagulation factor consumptive bleeding condition typical of overt DIC, but instead more closely resembles hypercoagulable thrombosis. This coagulopathy appears to be a core pathophysiology of COVID-19 as rising D-dimer levels, correlate with a poor prognosis, as do rising levels of IL-6 and CRP. IL-6 levels have been correlated to fibrinogen levels in one study, possibly through the acute phase reactant response (Ranucci et al., 2020). The pathogenesis of microthrombosis of the lung in COVID-19 is not known. There are multiple working hypotheses concerning this finding currently being assessed (Ackermann et al., 2020). Damage to the vascular endothelial glycocalyx can be caused by TNF-α, ischemia and bacterial lipopolysaccharide. As well, activated mast cells release cytokines, proteases, histamine, and heparinase, which degrade the glycocalyx (Alphonsus and Rodseth, 2014) and may thereby contribute to microthrombosis. Disruption of the glycocalyx exposes endothelial cell adhesion molecules, triggering further inflammation, rolling and adhesion of white blood cells and platelets (Becker et al., 2010). Glycocalyx components measured in serum positively correlate with increased mortality in septic patients (Nelson et al., 2008). Other causes of hypercoagulability include direct damage to ACE2 positive endothelial cells by viral invasion or secondary damage from the inflammatory response to the infection. Mast cells release heparin which activates the contact system, producing plasmin and bradykinin. Plasmin activation could account for the singular rise in D-dimer levels. Activation of platelets also seems likely as part of the thrombo-inflammatory response but their precise role in thrombus formation remains to be elucidated (Jackson et al., 2019). A more complete understanding awaits further study.

**FIGURE 12:**
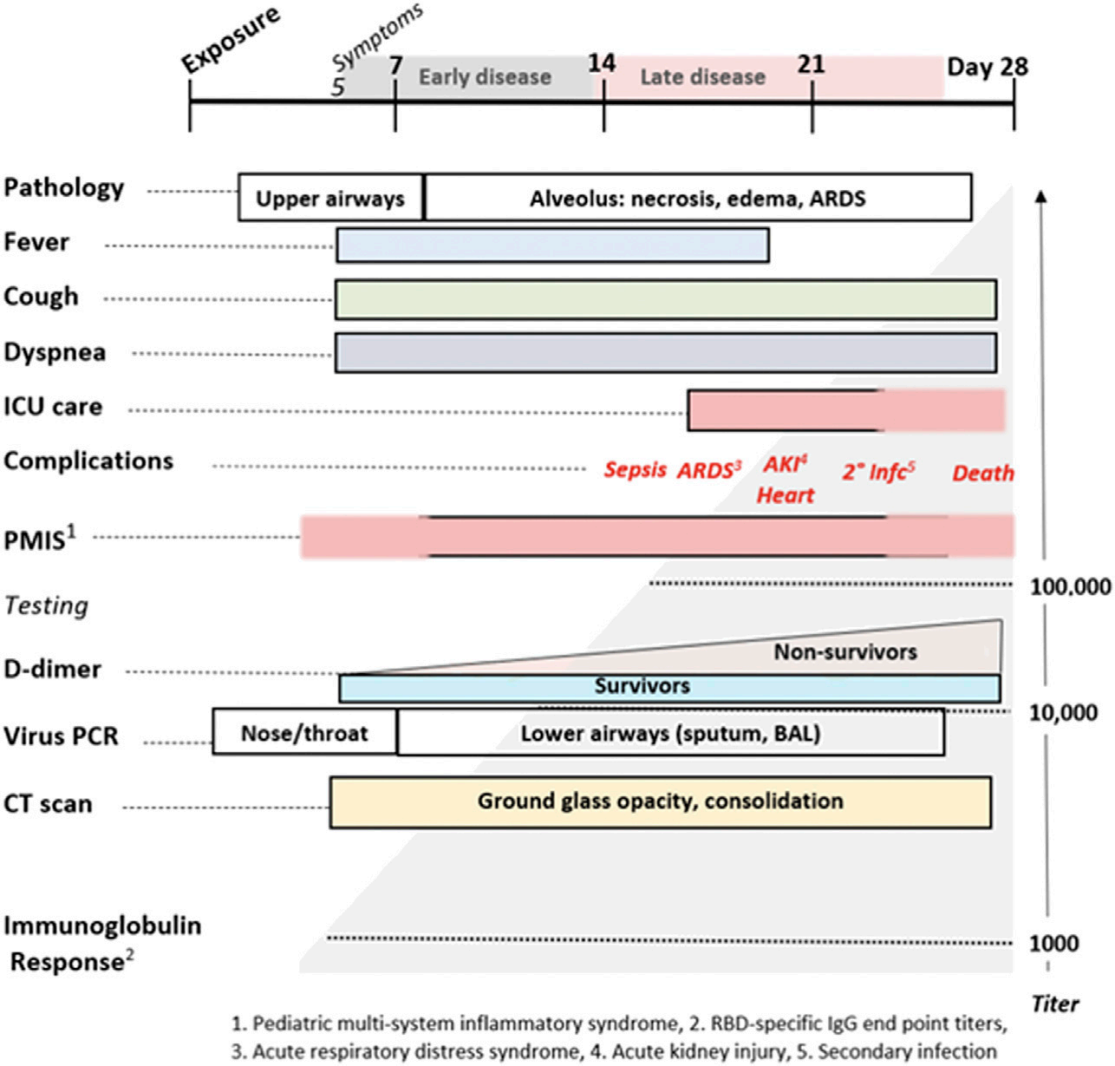
The Natural History of COVID-19. Modified from Oudkerk et al. (2020).

In addition to the usual features of a viral infection, early COVID-19 often presents with anosmia, ageusia, skin rashes including pruritis and urticaria, neuropsychiatric symptoms (including altered dream states), and silent hypoxia. These symptoms are all consistent with histamine signaling. Anosmia, ageusia, and other symptoms relating to cachexia are often reported in both COVID-19 and mast cell degranulation syndrome, and the potential role of histamine signaling in driving the pathophysiology of cachexia has been reviewed (Becker et al., 2012; Zwickl et al., 2019). As summarized in Figure 12, the distinctive later findings of abnormal coagulation, involvement of other organ systems and ARDS occur in the second week after the appearance of symptoms. This is coincidental with a rising immunoglobulin response to SARS-CoV-2 antigens. For a subset of patients, disease progress may suddenly worsen at days 7–10, and this correlates with the onset of SARS-CoV-2 spike protein neutralizing antibody titers (Suthar et al., 2020). In this study, it was shown that IgG starts to rise within 4 days post-symptoms, inconsistent with a first antigenic exposure (Suthar et al., 2020). Rapid onset of specific neutralizing antibody responses beginning less than seven days after exposure to SARS-CoV-2 implies a recall rather than primary B cell response, and therefore the response is being driven by a pre-existing memory cell population. SARS-CoV-2-reactive CD4^+^ T cells have been detected in ∼40–60% of unexposed individuals, suggesting cross-reactive T cell recognition between circulating ‘common cold’ coronaviruses and SARS-CoV-2 (Grifoni et al., 2020). These memory cells may have been educated by prior exposure to another coronavirus (e.g. circulating alphanumeric coronaviruses), raising concerns that this second phase of COVID-19 disease progression could share an immunologic basis with Dengue hemorrhagic fever (Mongkolsapaya et al., 2003). If pre-existing cross-reactive IgE “common cold” antibodies and/or associated memory B cell populations are also present, this may help account for or otherwise exacerbate dysfunctional mast cell degranulation. Antibodies produced from this early rapid humoral response may also drive further mast cell degranulation. During this phase rising D-dimer levels correlate with poor prognosis, as do measured levels of CRP and IL-6.

Current reviews seek to explain COVID-19 clinical and pathologic findings based on standard models of antiviral innate and adaptive immune responses which do not consider the potential role of mast cell activation and degranulation. Reviews emphasize the inflammatory cell response cascade associated with monocytes, macrophages (Merad and Martin, 2020), and adaptive T and B cell helper and effector responses (Vabret et al., 2020). These types of immune responses are also invoked to explain the novel microvascular pulmonary intravascular coagulopathy associated with COVID-19 (McGonagle et al., 2020).

We propose an alternative paradigm; SARS-CoV-2 infection-induced mast cell activation could account for some of the core pathologic cascade and much of the unusual symptomatology associated with COVID-19 (Kritas et al., 2019). Many of the unique clinical symptoms observed during the early phase of COVID-19 are consistent with known effects of histamine release (Conti et al., 2020). Histamine may act as an autocrine regulator of mast cell cytokine and TNF-a release in a PGE2-dependent fashion and based on *in vitro* studies the autocrine feedback appears to be mediated by H2 and H3 (Bissonnette, 1996). This model is consistent with the histopathologic findings seen at surgery, autopsies, and is supported by clinical pharmacologic findings suggesting potential benefits of histamine H_2_ receptor blockade using famotidine. This model is also supported by the significant overlap in the clinical signs and symptoms of the initial phase of COVID-19 disease and those of mast cell activation syndrome (MCAS) (Afrin et al., 2020; Butterfield, 2020; Weiler, 2020; Weinstock et al., 2020) as well similarities to Dengue hemorrhagic fever and shock syndrome (including T cell depletion) during the later phase of COVID-19 (Mongkolsapaya et al., 2003; Guzman and Harris, 2015; Redoni et al., 2020). The cardiac events, stroke, and related outcomes associated with COVID-19 also appear consistent with the Kounis syndrome (Gonzalez-de-Olano et al., 2011; Kounis, 2016; Kounis et al., 2020). This hypothesis is supported by the findings herein of increased numbers of mast cells in the alveolar septal walls and pulmonary parenchyma of SARS-CoV-2 infected AGM, as well as in the alveolar septa of COVID-19 patients (Motta Junior et al., 2020).

If COVID-19 is partially driven by dysfunctional mast cell degranulation, then a variety of medical interventions employing marketed drugs useful for treating mast cell-related disorders may help to reduce death and disease associated with SARS-CoV-2 infection. Examples include drugs with mast cell stabilizing activity such as beta-2 adrenoceptor antagonists (Scola et al., 2004) or cromolyn sodium (Zhang et al., 2016; Han et al., 2016), other histamine antagonists (for example H_1_ and H_4_ types) (Okayama et al., 1994; Marone et al., 2003; Hogan et al., 2020), leukotriene antagonists and leukotriene receptor antagonists (Fidan and Aydogdu, 2020), anti-inflammatory agents such as those developed for inflammatory bowel diseases, and mast cell activation inhibitors (Castells and Butterfield, 2019; Theoharides et al., 2019). If such repurposed drugs are used in combination with pharmaceuticals that directly inhibit SARS-CoV-2 infection or replication, it may be possible to rapidly develop potent, safe and effective outpatient treatments for preventing or treating COVID-19 until such time as a safe and effective SARS-CoV-2 vaccine becomes available.

**TABLE 2:** Summary of multiple working hypotheses tested

Hypothesis	Testing approach	Outcome
Does Famotidine act by binding or otherwise inhibiting SARS-CoV-2 Pl-Pro?	Direct PL-pro binding assay	Reject hypothesis
Does Famotidine act by binding or otherwise inhibiting SARS-CoV-2 M-Pro?	Literature review, prior studies	Reject hypothesis
Does Famotidine directly inhibit SARS-CoV-2 infection or replication in Vero cells?	TCID-50 viral inhibition assay	Reject hypothesis
Does Famotidine act via binding to Sigma 1 or 2 receptors	Sigma-1 and sigma-2 competition binding assays	Reject hypothesis
Is Famotidine selective for histamine H2 GPCR? Does Famotidine act via an off-target activity involving a G-coupled protein receptor (GPCR) other than the histamine H2 GPCR?	GPCRome screening	Reject hypothesis
Is Famotidine selective for histamine H2 GPCR? Does Famotidine act by blocking (antagonist or inverse agonist) histamine H1, H2, H3 or H4 GPCR receptor(s)?	GloSensor cAMP assays	Reject hypothesis for histamine H1 GPCR
		Accept hypothesis for histamine H2 GPCR
		Reject hypothesis for histamine H3 GPCR
		Reject hypothesis for histamine H4 GPCR
Does reported lack of beneficial clinical COVID-19 activity of histamine H2 blocking agent Cimetidine at standard GERD doses refute histamine H2 receptor MOA for Famotidine?	Comparative pharmacokinetic analysis	Reject hypothesis. GERD dose of Cimetidine does not provide sufficient systemic blood and tissue levels to block known tissue H2 receptor targets
Does standard GERD dose famotidine achieve sufficient systemic drug levels for beneficial clinical COVID-19 activity?	Pharmacokinetic analysis and literature review	Reject hypothesis. Concentrations required to mitigate effects on leucocyte targets exceed systemic tissue levels achieved via recommended GERD oral dosage
Does high dose famotidine administration affect the clinical course of COVID-19 disease?	Case study	Limited support for hypothesis, requiring further testing

## Data Availability

The raw data supporting the conclusion of this article will be made available by the authors, without undue reservation.
